# Microbial communities of the Laurentian Great Lakes reflect connectivity and local biogeochemistry

**DOI:** 10.1111/1462-2920.14862

**Published:** 2019-12-02

**Authors:** Sara F. Paver, Ryan J. Newton, Maureen L. Coleman

**Affiliations:** ^1^ Department of the Geophysical Sciences University of Chicago Chicago IL USA; ^2^ School of Freshwater Sciences, University of Wisconsin‐Milwaukee Milwaukee WI USA

## Abstract

The Laurentian Great Lakes are a vast, interconnected freshwater system spanning strong physicochemical gradients, thus constituting a powerful natural laboratory for addressing fundamental questions about microbial ecology and evolution. We present a comparative analysis of pelagic microbial communities across all five Laurentian Great Lakes, focusing on Bacterial and Archaeal picoplankton characterized via 16S rRNA amplicon sequencing. We collected samples throughout the water column from the major basins of each lake in spring and summer over 2 years. Two oligotypes, classified as LD12 (*Alphaproteobacteria*) and acI‐B1 (*Actinobacteria*), were among the most abundant in every sample. At the same time, microbial communities showed distinct patterns with depth during summer stratification. Deep hypolimnion samples were frequently dominated by a *Chloroflexi* oligotype that reached up to 19% relative abundance. Stratified surface communities differed between the colder, less productive upper lakes (Superior, Michigan, Huron) and warmer, more productive lower lakes (Erie, Ontario), in part due to an *Actinobacteria* oligotype (acI‐C2) that averaged 7.7% of sequences in the lower lakes but <0.2% in the upper lakes. Together, our findings suggest that both hydrologic connectivity and local selective pressures shape microbial communities in the Great Lakes and establish a framework for future investigations.

## Introduction

Large lakes are key to understanding the unifying principles that govern aquatic ecosystems. Large lakes are less influenced by their catchment than smaller lakes and experience ocean‐like physical processes, including strong currents and upwelling (Janssen *et al*., 2014). Among macroorganisms, fish exhibit strikingly similar life histories in large lakes and marine systems (Janssen *et al*., 2014; Pritt *et al*., 2014). Considering aquatic habitats along a continuum of temporal and spatial scales has the potential to uncover patterns that are not apparent under the traditional dichotomous view separating marine and freshwater systems (Janssen *et al*., 2014). Microbial communities represent a critical component of a cross‐scale aquatic ecosystem framework, yet to date, the microbial communities of large lakes have been far less studied compared to their small‐lake and marine counterparts.

The Laurentian Great Lakes are a unique system for exploring the processes that create and maintain microbial diversity in aquatic systems. Lakes Superior, Michigan, Huron, Erie and Ontario are part of an interconnected hydrologic system that covers over 244 000 km^2^ in collective surface area. Hydrologic connectivity combined with shipping traffic provides opportunities for microorganisms to disperse throughout the system. The Great Lakes are further defined by strong physical and chemical gradients, leading to striking differences in nutrient availability and primary productivity across lakes. Primary production in oligotrophic Lake Superior is lower than Station ALOHA in the North Pacific and on par with non‐bloom conditions in the Sargasso Sea (Hecky *et al*., 2004; Paytan and McLaughlin, 2007; Sterner, 2010) while cyanobacterial harmful algal blooms are a regular occurrence in the mesotrophic western basin of Lake Erie (Steffen *et al*., 2014). Temperature varies through the water column during summer stratification as well as across lakes; stratified surface waters of Lake Superior are frequently 8–14°C colder than the surface waters of Lake Erie (http://coastwatch.glerl.noaa.gov). Invasive species also have differentially impacted the five lakes. Dreissenid mussels, for example, are considered the primary stressor in Lakes Michigan, Erie and Ontario and have the least influence on Lake Superior (Smith *et al*., 2015). These invasive mussels are ecosystem engineers whose high filtering capacity has led to the collapse of the spring phytoplankton bloom in Lake Michigan (Fahnenstiel *et al*., 2010) and has altered internal phosphorus cycling (Hecky *et al*., 2004).

Our current understanding of the Great Lakes system as a whole has a microbial blindspot. As part of the U.S. Environmental Protection Agency's long‐term monitoring program, water quality, chlorophyll *a*, phytoplankton, zooplankton and benthos have been surveyed across all five lakes in the spring and summer of each year since 1992 (Barbiero *et al*., 2018). Notably, with the exception of cyanobacteria, bacterioplankton and archaeoplankton have not been captured by these surveys. Instead, most of what we know about Great Lakes microorganisms comes from local studies in one or a few of the lakes. These studies have begun to document taxonomic distributions with depth (Denef *et al*., 2015; Rozmarynowycz *et al*., 2019), between lakes with contrasting trophic states (Mukherjee *et al*., 2016; Olapade, 2018; Rozmarynowycz *et al*., 2019), along nearshore to offshore transects (Mueller‐Spitz *et al*., 2009; Newton and McLellan, 2015; Fujimoto *et al*., 2016), and seasonally (Fujimoto *et al*., 2016; Berry *et al*., 2017a). Furthermore, microbial community composition in Lake Erie appears to be related to the extent of winter ice cover (Beall *et al*., 2016) and the progression of cyanobacterial harmful algal blooms (Berry *et al*., 2017a). One of the only studies to compare microbial communities across all five lakes, before sequencing was widely available, found high similarity by DNA–DNA hybridization in epilimnetic picoplankton across the lakes but strong differences between the epilimnion and hypolimnion at a single station (Pascoe and Hicks 2004).

Here, we present results from the first comprehensive microbial survey of pelagic habitats in all five Laurentian Great Lakes, focusing on small planktonic Bacteria and Archaea (0.22–1.6 μm). We chose this size range because essentially all cells in this fraction are free‐living, i.e. not attached to particles, and therefore its composition is less sensitive to heterogeneity in particle availability (Fujimoto *et al*., 2016), facilitating comparisons across large spatial and temporal scales. Our objectives were to characterize patterns in microbial community structure across lakes and depths and to explore the selective pressures and neutral processes that underlie these patterns. We collected samples from depths spanning the surface to near bottom, at stations in each of the lakes' major basins, in the spring following ice‐off and in August during summer stratification over 2 years (Table S1). Our results lay the foundation for documenting community changes in the Great Lakes and for testing hypotheses about the drivers and implications of microbial diversity in this extensive, interconnected and biogeochemically varied freshwater system.

## Results

**Figure 1:**
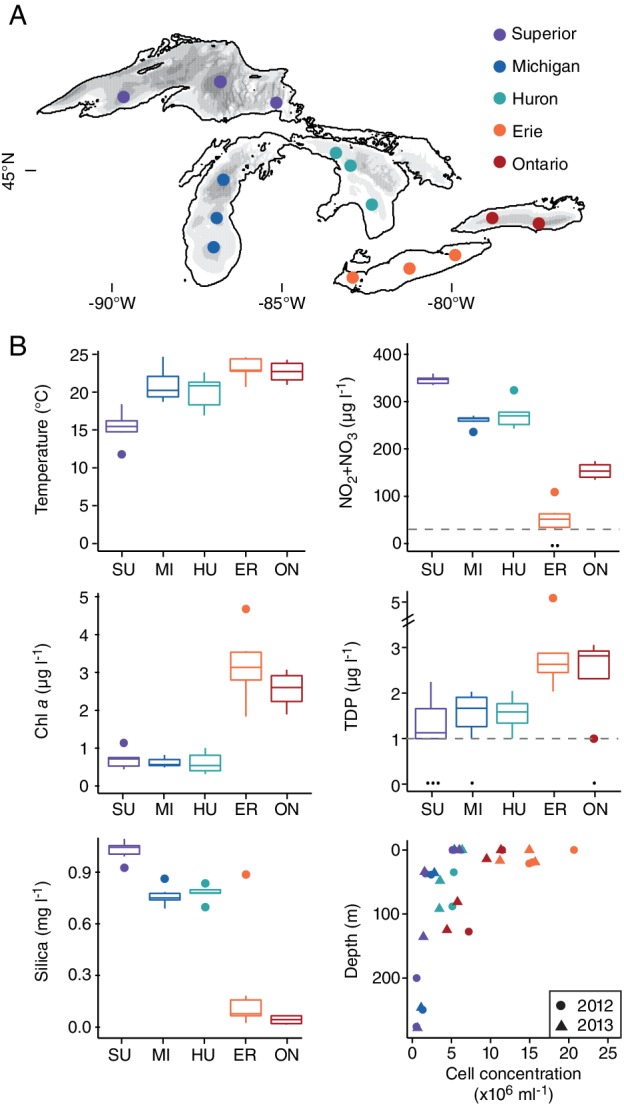
The Laurentian Great Lakes study system. A. Map of the lakes with grayscale illustrating depths from <100 m to >300 m. Points coloured by lake indicate the locations of sampling stations. B. Physicochemical parameters and bacterial cell counts across lakes. Temperature, chlorophyll *a* (chl *a*), dissolved SiO_3_/SiO_4_ as Si, nitrite plus nitrate (NO_2_^−^ + NO_3_^−^) and total dissolved phosphorus (TDP) are shown for all surface samples in 2012 and 2013 (average ± SE; data from US EPA Great Lakes Environmental Database). For N and P, black dots near the *x*‐axis indicate concentrations below the detection limit, which is indicated by a grey dashed line. These values were set to the detection limit for calculating mean/SE. Microbial cell concentrations as a function of depth are provided for one master station per lake (indicated in Table S1) in 2012 and 2013. Box plots and scatter points are colour coded by the lake.

Microbial communities from 154 samples across the Great Lakes (Fig. 1, Table S1) were characterized via amplicon sequencing of the V4 and V4‐V5 variable regions of the 16S rRNA gene [V4 region primers: 515F‐C, 806R‐H (Caporaso *et al*., 2012); V4‐V5 region primers: 515F‐Y, 926R (Parada *et al*., 2016)]. Sequences were analysed by minimum entropy decomposition (MED), an unsupervised oligotyping approach that uses Shannon entropy to delineate sequence groups called ‘oligotypes' that differ by as little as a single base pair (Eren *et al*., 2013, 2015). MED was applied to 13 817 282 and 21 012 923 sequences for the V4 and V4‐V5 data sets respectively, resulting in 2114 and 6360 oligotypes that accounted for an average of 96% and 91% of total microbial sequences. Although certain taxa were differentially represented in the V4 and V4‐V5 data sets likely due to primer bias [Tables S2 and S3; e.g., the V4 region primer set is biased against the SAR11 clade, which includes LD12; (Parada *et al*., 2016)], overall patterns in community composition were generally consistent between the two data sets (Fig. S1, Table S4). For simplicity, we focus on results from the 16S rRNA V4‐V5 region.

### *Depth is the primary factor structuring microbial communities*

**Figure 2:**
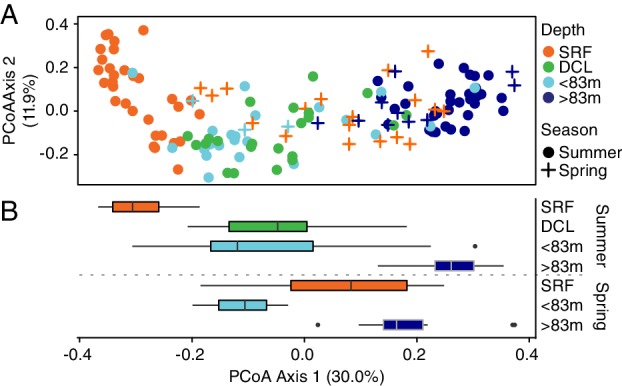
Microbial communities were strongly structured by depth during summer stratification. A. Principal coordinate analysis of pairwise Bray–Curtis similarities calculated between samples collected at several depths from each of the five Great Lakes during spring and summer surveys. Variation explained by each axis is indicated in parentheses. B. Boxplots of principal coordinate axis 1 values (median ± quartiles) for surface (SRF), deep chlorophyll layer (DCL), shallow hypolimnion (<83 m) and deep hypolimnion (>83 m) samples, highlighting differences observed across depths.

Using the V4‐V5 region data set, we examined how microbial communities are structured with depth and season, and spatially within and across the five Laurentian Great Lakes. In summer, the water column becomes thermally stratified, accompanied by the development of vertical structure in depth profiles of nutrients, dissolved oxygen and productivity (Figs S2 and S3). Accordingly, microbial communities were distinct across depths (Fig. 2, PERMANOVA: ‘depth’ pseudo‐*F* = 44.5, *R* ^2^ = 0.22, *p* < 0.001; ‘lake’ pseudo‐*F* = 9.0, *R* ^2^ = 0.18, *p* < 0.001, ‘depth*lake’ pseudo‐*F* = 4.6, *R* ^2^ = 0.09, *p* < 0.001). Physicochemical factors correlating with variation in microbial community composition included temperature (envfit *R* ^2^ = 0.79, *p* < 0.001), light availability (envfit *R* ^2^ = 0.77, *p* < 0.001) and total oxidized nitrogen (envfit *R* ^2^ = 0.63, *p* < 0.001) (Figs S4 and S5). During spring mixing, microbial communities were less distinct across depths, and spring surface communities resembled those of deeper summer communities (Fig. 2). At a given station, spring surface and bottom communities were very similar in composition, except in instances where the water column was not isothermal due to inverse stratification (e.g., Lake Superior stations in April 2013; Fig. S2).

**Figure 3:**
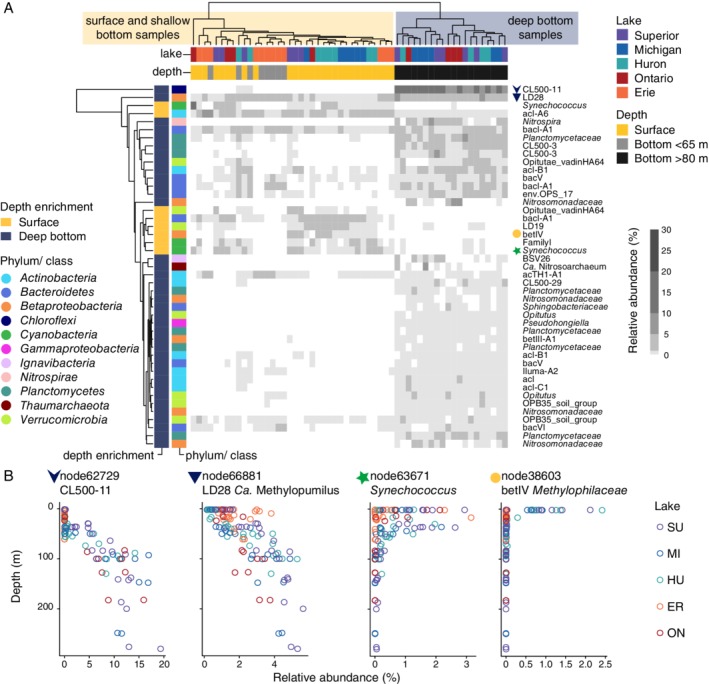
Depth‐stratified oligotypes exhibited a range of distribution patterns and included many deep‐water specialists. A. Heat map showing relative abundance of abundant oligotypes with significant depth enrichment, based on a Wald test comparing log2 fold change with *p* < 10^−6^. Dendrograms depict complete‐linkage clustering among samples and oligotypes, grouping together samples with similar oligotype composition and oligotypes with similar distributions across samples. B. Example depth distributions of four oligotypes during summer stratification: *Chloroflexi Anaerolineaceae* node62729, *Ca*. Methylopumilus LD28 node66881, *Cyanobacteria Synechococcus* node63671 and *Methylophilaceae* betIV node38603.

Owing to each lake's distinct bathymetry, our hypolimnion samples span a range of depths from 40 to 280 m [excluding the deep chlorophyll maximum layer (DCL) and shallow Lake Erie stations], and the hypolimnion microbial communities we observed show corresponding depth‐related variation. A characteristic deepwater assemblage was consistently observed in samples collected below 83 m, environments distinguished by a combination of temperatures <5°C and <0.5% of surface light availability (determined using MODIS‐AQUA estimates of light attenuation at 490 nm; see Methods). Phyla enriched in these deep waters relative to surface habitats included *Chloroflexi*, *Nitrospirae* and *Planctomycetes* (Table S5). At the oligotype level, many deep‐water enriched taxa were rare in surface waters and consistently observed in samples collected near the bottom of the deepest stations (Fig. 3). The most striking deep‐water specialist oligotype was identified to the *Anaerolineaceae* family of *Chloroflexi* and shared 99.7% identity to the CL500‐11 16S rRNA gene from Crater Lake (Urbach *et al*., 2001). This CL500‐11 oligotype was nearly absent in surface samples yet consistently observed across all samples collected below 62 m deep, comprising 6%–19% of sequences for samples collected below 100 m (node62729; Fig. 3B). In contrast, other deep‐enriched oligotypes were observed throughout the water column and exhibited more gradual changes in relative abundance with depth (e.g., *Ca*. Methylopumilus/LD28 node66881; Fig. 3B).

Communities observed at the surface of stratified water columns in August had a distinct composition that re‐assembled each year following the onset of stratification. This surface community lacked deep‐water specialist taxa and was enriched in other specific taxa (Fig. 3). *Synechococcus* oligotypes were found at high relative abundances in both surface and DCL samples (e.g., node63671; Fig. 3B). Additionally, a *Methylophilaceae* betIV oligotype exhibited extreme surface enrichment, detected only in surface samples from the upper lakes (node38603; Fig. 3B).

### *Upper and lower lakes harbour distinct surface communities*

We next asked whether communities differed significantly across lakes, focusing on summer surface samples in order to remove the predominant depth effect. Community composition differed between the upper lakes (Superior, Michigan and Huron) and the lower lakes (Erie and Ontario) (Fig. S6; ‘upper vs. lower lake’ PerMANOVA: pseudo‐*F* = 11.2, *R* ^2^ = 0.31, *p* < 0.001; ‘Lake’ PerMANOVA: pseudo‐*F* = 5.6, *R* ^2^ = 0.50, *p* < 0.001), which mirrors differences in productivity and other factors (Fig. 1B). Because environmental variables covary strongly across the Great Lakes, many measured parameters correlated with microbial community composition across lakes, including total oxidized nitrogen (envfit *R* ^2^ = 0.86, *p* < 0.001), chlorophyll *a* (envfit *R* ^2^ = 0.78, *p* < 0.001), temperature (envfit *R* ^2^ = 0.77, *p* < 0.001), and dissolved silica (envfit *R* ^2^ = 0.70, *p* < 0.001) (Fig. S6).

**Figure 4:**
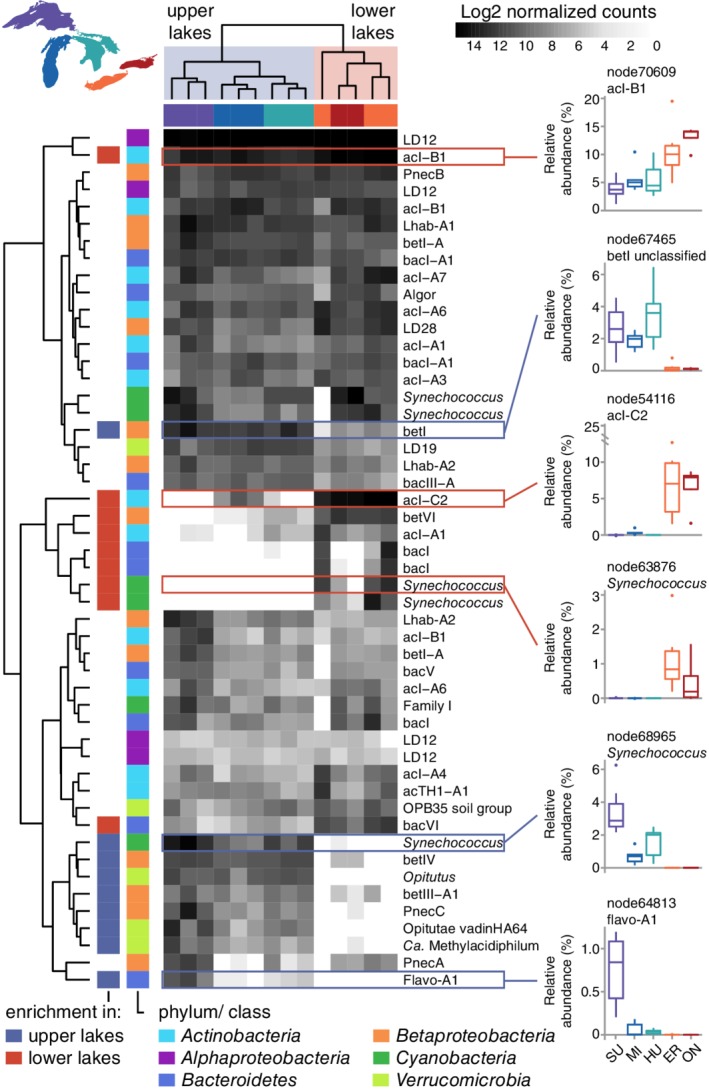
Surface communities across the Great Lakes are comprised of both ubiquitous and region‐specific taxa. Heat map shows the 50 most abundant oligotypes during summer 2013, as log2 transformed counts normalized for sequencing depth. Dendrograms depict complete‐linkage clustering among samples (top) and oligotypes (left). Samples, colour coded by lake, formed two groups: upper lakes (Superior, Michigan and Huron) and lower lakes (Erie and Ontario). Oligotype clusters generally reflect ubiquitous taxa or taxa enriched in upper or lower lakes. The leftmost colour bar indicates oligotypes that were significantly enriched in upper or lower lakes based on a Wald test comparing log2 fold change with *p* < 10^−6^. The second colour bar identifies the phylum or proteobacterial class of each oligotype. Relative abundance by lake (mean ± interquartile range) is summarized for select oligotypes with region‐specific distributions.

Some oligotypes in surface waters exhibited a clear preference for upper or lower lakes, while many others showed no lake preference (Fig. 4). Among *Synechococcus*, distinct oligotypes were enriched in upper or lower lakes (e.g., node63876 and node68965; Fig. 4), coexisting with several others that were widespread across lakes. Upper lakes were strongly enriched in several *Betaproteobacteria* (e.g., node67465; Fig. 4) and *Verrucomicrobia* oligotypes, while lower lakes were enriched in oligotypes representing several *Actinobacteria* lineages. One of the most conspicuous oligotypes differentiating upper and lower lakes, belonging to the acI‐C2 lineage of freshwater *Actinobacteria*, comprised an average of 7.7% of microbial sequences from Lake Erie and Ontario surface samples but was not abundant in the upper lakes (node54116; Fig. 4).

### *Ubiquitous and habitat‐specialist taxa coexist in high abundance*

**Figure 5:**
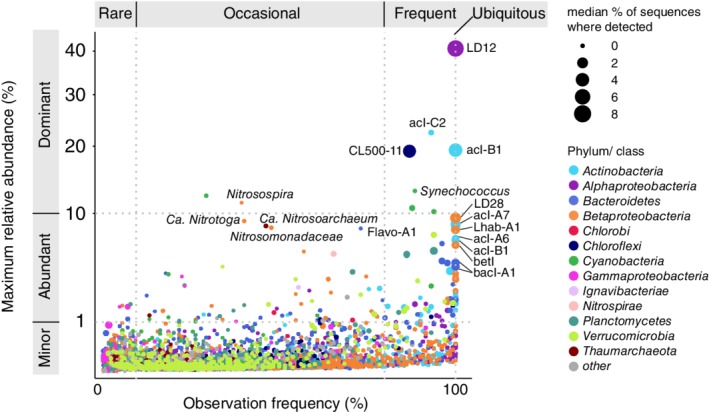
Maximum relative abundance of each oligotype as a function of the percent of samples where that oligotype was detected. Oligotypes are colour‐coded by phylum. Included in this analysis are samples collected in spring and summer from surface, deep chlorophyll layer (summer, if present), mid‐hypolimnion (summer) and bottom depths. See Fig. S7 for this analysis carried out separately for specific sets of samples. Symbol size corresponds to the median proportion of sequences for each oligotype (when detected).

Nine oligotypes reached dominant relative abundance (>10% of sequences) in at least one sample, representing three broad patterns of distribution. Two of the nine, classified as LD12 and acI‐B1, were both abundant and ubiquitous, detected in every sample with median relative abundances of 8.6% and 5.2% respectively. A second group included oligotypes that were frequently detected across samples but were enriched in specific habitats (Fig. 5, Figs S7 and S8): deep‐specialist *Chloroflexi* (Fig. 3), photic zone specialist *Synechococcus* (Fig. 3) and lower lake‐specialist acI‐C2 (Fig. 4). A third group, consisting of oligotypes of *Nitrosospira* and *Synechococcus*, was detected less often but achieved very high abundance in some samples. A similar ‘bloomy’ distribution—i.e. sporadic but sometimes very abundant—was also observed for the nitrifying taxa *Ca*. Nitrotoga, *Ca*. Nitrosoarchaeum and *Nitrosomonadaceae* (Fig. 5).

**Figure 6:**
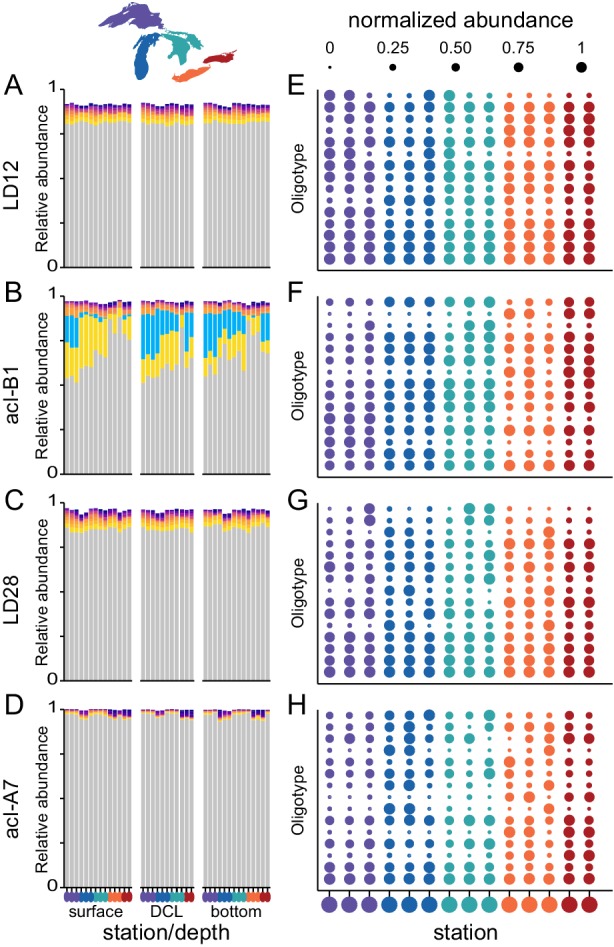
Manual oligotyping of four tribes revealed differences in composition with depth and across lakes in summer 2013. A–D. Stacked bar plots with the 10 most abundant oligotypes collected at three depths for each station. E–H. Bubble plots depict the distribution of the 15 most abundant oligotypes within each tribe across surface samples, ordered by rank abundance (most abundant at bottom). Relative abundances (proportion of total tribe sequences) were normalized within each oligotype (i.e., 1 corresponds to the highest relative abundance observed for that oligotype across samples, 0.25 corresponds to a relative abundance that is 25% of the highest observed relative abundance). Stations are colour coded by lake and ordered from north to south in Michigan and west to east in the other lakes.

The ubiquitous oligotypes that we detected in every sample are representatives of globally abundant freshwater lineages, and we wondered how much diversity existed in these extremely successful groups. We focused on four major freshwater tribes: LD12, acI‐B1, LD28 and acI‐A7 (Fig. 6). A tribe is the most refined and species‐approximating unit of taxonomy for freshwater bacteria based on 16S rRNA gene sequences, consisting of sequences that cluster as a monophyletic branch with every sequence having at least 97% identity to another sequence within that branch (Newton *et al*., 2011). For each tribe, we performed supervised oligotyping, which is based on the same principles as unsupervised oligotyping (MED) but is potentially more sensitive due to manual inspection and curation (Eren *et al*., 2013). Both MED and supervised oligotyping yielded similar patterns of diversity for each tribe (data not shown). Each tribe could be resolved into many constituent oligotypes, ranging from 17 for acI‐A7 to 101 for LD12 (Table S6). Despite this high oligotype diversity, each tribe was dominated by a single oligotype, which was dominated by a single unique sequence that accounted for 49%–68% of all sequences within that tribe (Table S6). Minor within‐tribe oligotypes were generally not abundant, comprising up to 3.3% of sequences within their respective tribe across all samples. There were two exceptions: in tribe acI‐B1, minor oligotypes reached up to 26% and 29% of sequences (yellow and blue bars, Fig. 6B, Fig. S9). Oligotype composition within each tribe varied with lake and depth (PerMANOVA; Table S6), reflecting the fact that some minor oligotypes showed habitat specificity even though the major oligotype in each tribe was generally ubiquitous (Fig. 6). Some minor oligotypes even showed patchy basin‐specific distributions within a lake, i.e. were abundant at only one or two stations in a given lake (Fig. 6E‐H).

## Discussion

### *Signatures of system‐wide connectivity*

A striking feature of microbial communities in the Laurentian Great Lakes is the ubiquitous occurrence of some oligotypes, and even individual amplicon sequence variants, across the entire system (Figs 5 and 6, Table S6). Previous findings have suggested strong genetic connectivity between *Microcystis* in lakes Erie and Ontario (Davis *et al*., 2014), and an earlier study suggested genetic similarity across lakes based on DNA–DNA hybridization (Pascoe and Hicks 2004); our results go further in showing that dozens of oligotypes spanning *Proteobacteria*, *Actinobacteria* and *Bacteroidetes* are ubiquitous across the biogeochemical extremes of the Great Lakes. Moreover, individual sequence variants of two lineages, acI‐B1 and LD12, are among the most abundant sequences in every sample. By contrast, microbial communities across 10 unconnected lakes in Japan showed more lake‐specific composition (Okazaki *et al*., 2017). The prevalence of ubiquitous taxa in our study suggests that microbial picoplankton are not dispersal limited across open waters of the Great Lakes and that the habitats we sampled share some broad characteristics that support these dominant taxa. The lineage acI‐B1, for example, tends to prevail in systems with relatively high pH and low dissolved organic carbon that is primarily autochthonous in origin (Jones *et al*., 2009; Newton *et al*., 2011)—characteristics that are shared by each of the Great Lakes (Phillips *et al*., 2015; Zhou *et al*., 2016). Notably, all of the samples presented here were collected at offshore stations; nearshore stations may be dominated by different taxa, as documented in southern Lake Michigan (Newton and McLellan, 2015; Fujimoto *et al*., 2016) and northern bays of Lake Superior (Rozmarynowycz *et al*., 2019).

Our ability to detect signatures of selection, dispersal, neutral processes and mutation on microbial communities depends on the phylogenetic scale (Hanson *et al*., 2012; Ladau and Eloe‐Fadrosh, 2019). The findings presented here are based entirely on a single, slowly evolving locus, the 16S rRNA gene. However, there are many cases where microorganisms that are indistinguishable by 16S rRNA gene amplicons are known to have distinct gene content, physiological properties and ecological distributions (e.g., Jaspers and Overmann, 2004; Thompson *et al*., 2005; Hahn *et al*., 2016; Garcia *et al*., 2018), and ecologically important traits are often uncoupled from 16S rRNA‐based taxonomic units (e.g., Martiny *et al*., 2006, 2012; Berry *et al*., 2017b). Therefore, it is likely that there are finer‐scale patterns of diversity across the Great Lakes that are obscured by the limited phylogenetic resolution of the 16S rRNA gene. It is possible, for example, that locally adapted subpopulations of dominant taxa (e.g., acI‐B1, LD12) occupy particular lakes or depths. Even streamlined genomes characteristic of LD12 and acI frequently harbour genomic islands or hypervariable regions, and these islands may facilitate adaptation to local nutrient availability, viral predation and other environmental pressures (Zaremba‐Niedzwiedzka *et al*., 2013; Neuenschwander *et al*., 2017). Recently, comparative genomics has highlighted the key role that genomic islands may play in ecological specialization among common freshwater *Polynucleobacter* populations (Hoetzinger *et al*., 2017). Hence, it is clear that 16S rRNA patterns do not tell the whole story, and the next challenge is to expand our phylogenetic lens to other marker genes and whole genomes.

### *Environmental drivers of microbial diversity across the Laurentian Great Lakes*

Although ubiquitous sequence types are a prominent feature of microbial communities in the Great Lakes, we also found strong community divergence, both across depths and across lakes. These patterns point to particular environmental factors that may be driving the distributions of some taxa. Communities were most strongly differentiated along the axis of depth, with characteristic assemblages appearing at the surface and the deepest samples (>83 m; Figs 2 and 3). This depth stratification of microbial communities is consistent with previous observations in the upper Great Lakes (Denef *et al*., 2015; Rozmarynowycz *et al*., 2019), in other deep lakes with oxygenated hypolimnia [e.g., Lake Baikal (Kurilkina *et al*., 2016), Lake Zurich (Salcher *et al*., 2011), Lake Biwa (Okazaki and Nakano, 2016)] and in the global ocean (Sunagawa *et al*., 2015). The deepest habitats that we surveyed are a freshwater analogue to the marine twilight zone, a mesopelagic layer beginning at the depth where 1% of incident light is available and ending where there is no light (~200–1000 m in the ocean). Similar to observations in the global ocean, we expect enrichment of photosynthesis in well‐lit surface waters of the Great Lakes relative to the twilight zone, while aerobic respiration, flagellar assembly, chemotaxis and nitrification are likely to be more prevalent in the twilight zone than in the surface (Sunagawa *et al*., 2015).

Light availability is a key selective pressure controlling the distribution of some taxa; yet light availability alone does not predict the composition of deep‐water assemblages across the Great Lakes. This is illustrated by the distribution of a Chloroflexi *Anaerolineaceae* oligotype [*Ca*. Profundisolitariaceae (Mehrshad *et al*., 2018)]—an abundant and characteristic member of deep‐water communities in the Great Lakes. CL500‐11 is ubiquitous and abundant in deep, oxygenated freshwater hypolimnia globally (Okazaki *et al*., 2017, 2018; Mehrshad *et al*., 2018; Rozmarynowycz *et al*., 2019). In the Great Lakes, a CL500‐11 oligotype was detected in all samples collected below 62 m and reached maximum relative abundances below ~100 m (Fig. 3B; node62729), suggesting that light availability, or a factor strongly correlated with light (e.g., competition with phototrophs; the composition and concentration of sinking organic matter), influences its distribution. While we rarely observed CL500‐11 in stratified surface samples (Fig. 3B), CL500‐11 has previously been detected in the surface mixed layer of Lake Superior's northern bays (Rozmarynowycz *et al*., 2019); these bays experience sediment resuspension and reduced light availability, consistent with a low‐light preference for this organism. A genome reconstruction for CL500‐11 from Lake Michigan contained genes for flagellar motility and rhodopsin, a light‐driven ion pump, which could enable cells to relocate in response to light (Denef *et al*., 2015).

At the same time, light availability does not explain the rarity of CL500‐11 in the eastern basin of Lake Erie, the bottom of which is well within the twilight zone (<0.01% of surface light availability). CL500‐11 is thought to be sensitive to low oxygen conditions (Okazaki *et al*., 2018), but the eastern basin of Erie is not severely oxygen‐depleted relative to deep basins in the other Great Lakes (9.1 and 10.1 mg l^−1^ vs. 11.3–13.4 mg l^−1^). The deepest basin of Lake Erie is also slightly warmer (~5.6°C vs. <4.8°C) than habitats with comparable light availabilities in the other lakes, but this temperature is well within the range where CL500‐11 is abundant in other systems (Okazaki *et al*., 2018). Hence, the light, temperature and oxygen conditions in eastern Erie would seem to favour CL500‐11. One possible explanation is that the abundance of CL500‐11 depends on additional factors not measured here (e.g., organic matter composition, predation). Physical processes are likely important as well. CL500‐11 cells may be filtered out by selection in the basins upstream, which are shallower, warmer, more productive, and, in the case of central Erie, experience seasonal hypoxia. Given Erie's short residence time (2.6 years), slow‐growing populations may not recover in the eastern basin, even if conditions are favourable.

Similarly, light alone does not explain the distribution of nitrifying lineages across the Great Lakes. Oligotypes representing nitrifier taxa, including *Nitrosospira*, *Ca*. Nitrotoga, *Ca*. Nitrosoarchaeum and *Nitrospira*, typically had a ‘bloomy’ distribution, abundant in certain deep samples (up to 5%–11%) but undetected or rare in other deep samples. Prior work has documented deep‐water enrichment of nitrifying taxa in the Great Lakes (Mukherjee *et al*., 2016), though we detected somewhat different taxonomic assemblages than prior FISH‐based studies (Small *et al*., 2013; Mukherjee *et al*., 2016). These discrepancies could be due to the smaller size fraction we examined (<1.6 μm), or to spatiotemporal heterogeneity in nitrifier distributions. Similarly, the patchy spatial distribution of nitrifiers we observed across oxygenated deep‐water habitats parallels findings from Japanese lakes (Okazaki *et al*., 2017) and could reflect strong temporal dynamics of nitrifiers, as observed in Lake Michigan (Fujimoto *et al*., 2016) and Lake Biwa (Okazaki and Nakano, 2016). Spatio‐temporal patchiness in nutrient availability and biotic factors (e.g., competition for ammonium, invasive mussels that supply reduced nitrogen) (Cuhel and Aguilar, 2013; Hugoni *et al*., 2013) may underlie the variability in abundance and taxonomic composition of nitrifier assemblages that we observed.

Nutrient availability and productivity also appear to influence community structure in well‐lit surface waters. We found surface community divergence between the cooler, less productive upper lakes (Superior, Michigan and Huron) and the warmer, more productive lower lakes (Erie and Ontario) (Fig. 4, Fig. S4). An oligotype classified to the actinobacterial acI‐C2 tribe was the most conspicuous oligotype differentiating the lower lakes from the upper lakes (node54116; Fig. 4). The acI‐C2 oligotype achieved the second‐highest maximum relative abundance of any oligotype across our data set and averaged 7.7% of sequences in Erie and Ontario surface samples while not being detected in some of the upper lake samples. Bacteria in the acI‐C lineage have been found to increase over the course of a cyanobacterial bloom in Lake Erie (Berry *et al*., 2017a). Although acI‐C2 has been observed in high abundance in some freshwater systems (Savio *et al*., 2015; Okazaki *et al*., 2017), distinguishing features of acI‐C2 physiology or ecology are unknown. Similar to other acI lineages, we expect acI‐C2 to have streamlined genomes that contain actinorhodopsin genes and benefit from catalase released into the environment as a public good by other bacteria (Kang *et al*., 2017; Kim *et al*., 2019).

A betIV oligotype (*Methylophilaceae* family, node38603; Fig. 3B) exhibiting extreme surface enrichment also helped to differentiate upper and lower lakes. High relative abundances of this oligotype (0.5%–2.4% of sequences), which has an identical 16S rRNA V4‐V5 gene sequence to *Ca*. Methylosemipumilus turicensis isolated from Lake Zurich (Salcher *et al*., 2015, 2019), were exclusive to upper lake surface samples collected in August of both years (Fig. 3B). Population dynamics in Lake Zurich coupled with genomic analysis suggest that *Ca*. Methylosemipumilus turicensis, like *Ca*. Methylopumilus planktonicus (LD28), uses C1 compounds released by phytoplankton (Salcher *et al*., 2015). Based on our results, we hypothesize that node38603 represents methylotrophs that rely on exudates released by phytoplankton whose abundance peaks at the surface during summer stratification. These methylotrophs or their phytoplankton associates may also have a specific temperature range or a dependence on nitrate (Kalyuhznaya *et al*., 2009), which could help explain their enrichment in the upper lakes (Fig. 1). These inferences are in contrast to Lake Zurich *Ca*. Methylosemipumilus turicensis, which generally blooms during the spring and whose abundance correlates with ammonium levels (Salcher *et al*., 2015), potentially due to differences in the phytoplankton upon which these populations depend.

Finally, temperature variation likely contributes to the complex distribution patterns of some taxa across lakes and depths. For example, one minor oligotype of acI‐B1 appears to be adapted to low temperatures, achieving comparatively high relative abundances (up to 26% of acI‐B1 sequences; blue bar in Fig. 6B) in spring versus summer surface samples, in summer surface waters of Lake Superior versus the other lakes, and below the thermocline of all lakes except Erie (Fig. S9). There is growing evidence to suggest that very closely related populations occupying different thermal niches undergo seasonal replacement (Eren *et al*., 2013; Ward *et al*., 2017) and/or partition the water column with depth (Okazaki *et al*., 2017; Garcia‐Garcia *et al*., 2019). Microorganisms may be responding to cross‐lake changes in temperature and other environmental conditions directly or through their interactions with phytoplankton (e.g., as appears to be the case for betIV node38603), similar to what has been described in north temperate humic lakes (Paver and Kent, 2017).

## Conclusions

The Laurentian Great Lakes encompass biogeochemical extremes, from the highly productive western basin of Lake Erie to the cold, dark depths of Lake Superior. Nevertheless, our findings reveal that a common set of microbial taxa is shared across these environments, suggesting unimpeded dispersal and broad similarities among the offshore stations we sampled. The apparent ubiquity of some taxa across this varied system begs new questions: how much genome‐wide diversity is contained within a single oligotype, and what processes have shaped this diversity? At the same time, other oligotypes show strong enrichment in certain locations, demonstrating an important role for selection in shaping these communities. In particular, our findings highlight a vast underexplored freshwater twilight zone—quadrillions of litres of perpetually cold and aphotic pelagic habitat that may foster novel metabolic, physiological and ecological adaptations. Together, the spatial patterns of microbial community diversity we describe provide a necessary foundation for exploring ecological and evolutionary responses to environmental change in this critical ecosystem. More broadly, we propose that the Laurentian Great Lakes are a powerful natural laboratory for understanding how processes such as dispersal and selection interact to shape the diversity of microbial genomes and communities.

## Methods

### *Sample collection*

We collected water samples from multiple stations in each of the five Laurentian Great Lakes (Fig. 1A, Table S1) aboard the R/V *Lake Guardian* during the U.S. Environmental Protection Agency's Spring and Summer Survey cruises in 2012 and 2013. At each station, the CTD/rosette sampler generated temperature, fluorescence, dissolved oxygen and turbidity depth profiles as it descended through the water column and collected water samples from specific depths as it ascended to the surface. For every sampling event, we collected water from the surface (~2 m) and 10 m from the bottom, with the exception of comparatively shallow stations in Lake Erie (11–60 m) where bottom samples were collected 1 m from the bottom. During Summer Surveys, the water column of each lake was stratified and we collected samples from two additional depths: the DCL (if present) and the middle of the hypolimnion, an intermediate depth between the upper hypolimnion and bottom of the lake. Water samples (4.5–8 L) were pre‐filtered through GF/A filters (Whatman) and microorganisms were concentrated onto 0.22 μm Millipore Sterivex filters (SVGP01050) in 2012 and 0.22 μm Millipore Express Plus filters (GPWP04700) in 2013 using a peristaltic pump. Filters were stored at −80°C prior to DNA extraction.

### *Environmental data*

Water chemistry (e.g., total oxidized nitrogen, total phosphorus) and chlorophyll *a* data were generated by the U.S. Environmental Protection Agency's Great Lakes National Program Office according to their standard protocols (GLNPO, 2010) and downloaded from the Great Lakes Environmental Database system, which can be accessed through the Environmental Protection Agency Central Data Exchange (https://cdx.epa.gov/). Light availability estimates were calculated using satellite imagery from NASA's Moderate Resolution Imaging Spectroradiometer MODIS‐Aqua diffuse attenuation coefficient at 490 nm (NASA Goddard Space Flight Centre, Ocean Biology Processing Group, 2014). The Aqua satellite provides colour data at 1 km resolution and temperature data at 4 km resolution; measurements were associated with one of our sampling sites if collection fell within 4 km and 2 days of sampling. Satellite water clarity data have been demonstrated to compare favourably with *in situ* measurements (Yousef *et al*., 2017).

Samples for microbial cell counts were preserved with 0.125% (final concentration) EM grade glutaraldehyde (Electron Microscopy Sciences), incubated at room temperature in the dark for 10 min, flash‐frozen in liquid nitrogen and stored at −80°C. Cells in preserved samples were thawed, stained with 0.5× SYBR Green (Thermo Fisher Scientific), diluted 3×–9× depending on initial concentrations and quantified using an Attune Acoustic Focusing Flow Cytometer. Particles were counted as cells if fluorescence emitted following excitation with 530/30 and 574/576 nm lasers exceeded background noise levels.

### *DNA extraction and sequencing*

We extracted DNA from filters using a modified Phenol:Chloroform extraction protocol (Wright *et al*., 2009). Filters were thawed on ice and incubated with 4 ml of lysis buffer (50 mM Tris pH 8.3, 40 mM EDTA, 0.75 M sucrose), 200 μl lysozyme solution (125 mg ml^−1^ in TE buffer; Sigma) and 40 μl RNase A solution (10 μg ml^−1^) in a rotating hybridization oven at 37°C for 1 h. We then added 200 μl Proteinase K solution (>600 mAU ml^−1^; Qiagen) and 200 μl 20% SDS (Sigma) to the filter and incubated the filters for 90 min in a rotating hybridization oven at 55°C. Lysate and a 1 ml lysis buffer rinse of the filter were added to a fresh tube, combined with an equal volume of Phenol:Chloroform:Isoamyl Alcohol (Sigma), vortexed to mix, and centrifuged at 2500*g* for 5 min. The top aqueous layer was transferred to a new tube, combined with an equal volume of Chloroform:Isoamyl Alcohol, and vortexed to mix. Tubes were centrifuged until the aqueous layer appeared clear. The aqueous layer was transferred into an Amicon Ultra‐4 centrifugal filter (10 000 kDa nominal cut‐off), centrifuged at 3500*g* to concentrate the DNA, washed 3× with 2 ml TE buffer and centrifuged until DNA was concentrated to 200–500 μl. DNA samples were further concentrated and purified by ethanol precipitation. DNA was quantified by fluorometry (Qubit, Thermo‐Fisher Scientific), diluted to 5 ng μl^−1^ and submitted for sequencing at the Joint Genome Institute. There, each sample was amplified according to their standard operating protocol (Daum, 2017) using two barcoded 16s rRNA primer sets: 515F‐C and 806R (V4) (Caporaso *et al*., 2012) and 515F‐Y and 926R (V4‐V5) (Parada *et al*., 2016). Amplicons were sequenced using 2 × 300 chemistry on an Illumina MiSeq sequencer. V4‐V5 region run 1, which included all summer 2012 samples, yielded 2.4–4.6 × 10^4^ sequences per sample, and run 2, which included all summer 2013 samples, yielded 1.2–2.6 × 10^5^ sequences per sample; V4 region runs yielded between 4.9 × 10^3^ and 1.5 × 10^5^ sequences.

### *Sequence processing*

We de‐interleaved the raw sequence reads using the bbtools reformat.sh script (available at https://sourceforge.net/projects/bbmap/) and processed paired reads using mothur version 1.38.1 and a modification of the MiSeq standard operating protocol accessed 23 May 2017 (Kozich *et al*., 2013). Briefly, forward and reverse reads were combined using make.contigs. For V4 region amplicons, we implemented the trimoverlap parameter because the 2 × 300 bp sequence reads exceeded the length of the v4 amplicon. Primers were removed using trim.seqs, allowing up to four differences to the primer sequences. Screen.seqs removed sequences containing ambiguities and those with a maximum length exceeding 275 or 389 for V4 and V4‐V5 region amplicons respectively. Unique screened sequences generated by unique.seqs were aligned to the Silva v. 128 reference alignment and further screened to only include sequences spanning the full length of each amplicon region. Skipping the pre.cluster step, we removed chimeras using the chimera.uchime function, classified sequences using the Silva database via the Wang method (Wang *et al*., 2007) and removed sequences classified as ‘chloroplast’, ‘mitochondria’, ‘unknown’ or ‘eukaryota’. Sequences were additionally classified using the freshwater TaxAss database (https://github.com/McMahonLab/TaxAss) (Rohwer *et al*., 2018). Sequences were delineated into operational taxonomic units using oligotyping, which employs Shannon entropy to iteratively group sequences using information‐rich nucleotide positions and discard stochastic variation (Eren *et al*., 2013, 2015). Unsupervised oligotyping, termed MED, was carried out separately for all Bacterial and all Archaeal sequences with a minimum substantive abundance (−M) of 50 and maximum variation allowed (−V) of 4 for each oligotype. Subsampling was not deemed necessary as sequence depths were within two orders of magnitude of each other (Eren *et al*., 2015). The resulting taxonomic units are referred to as ‘unsupervised oligotypes’ or ‘oligotypes’. Supervised oligotyping with a −M of 500 was carried out for sequences within specific taxonomic groups with resulting taxonomic units referred to as ‘supervised oligotypes’ or ‘within‐tribe oligotypes’. Sequences are available on the Joint Genome Institute's genome data portal (http://genome.jgi.doe.gov/; project identifiers, 1045074 and 1045077) and at NCBI (BioProject PRJNA591360).

### *Statistical analysis*

We analysed our data using R version 3.3.2 (R Core Team, 2016). All R code and associated data files, as well as fasta files corresponding to unsupervised and supervised oligotypes, are available at: https://bitbucket.org/greatlakes/gl_spatial_diversity.git. We used Phyloseq (version 1.19.1) to convert raw sequence counts to relative abundance, subset our data for various analyses, and visualize patterns (McMurdie and Holmes, 2013). We visualized community‐level patterns across the entire data set and across summer stratified samples using Principal Coordinate Analysis of Bray–Curtis dissimilarities calculated between each pair of samples. Physicochemical factors correlating with variation in microbial community composition as represented by principal coordinate axes were identified using the envfit function in the vegan package version 2.4‐1 (Oksanen *et al*., 2016). To test for effects of depth and lake on microbial community composition and the composition of oligotypes within specific freshwater bacterial tribes during summer stratification, we carried out Permutational Multivariate Analysis of Variance as implemented by the adonis function in the vegan package (Oksanen *et al*., 2016). We compared (a) microbial communities characterized using primers targeting the V4 region versus the V4‐V5 region, (b) oligotypes in surface versus deep bottom samples, (c) oligotypes in upper lakes versus lower lakes by testing for differences in log2 fold change of sequence count data using a parametric Wald test implemented by DESeq2 (Love *et al*., 2014).

## Supporting information


**Figure S1** Comparing two primer sets for 16S rRNA gene amplicon sequencing. (A, B) Rank abundance curves for all Great Lakes samples, based on (A) V4 region primers or (B) V4‐V5 region primers. Points are coloured by phylum/proteobacterial class. (C, D) Relative abundance estimates in the V4 data set vs. the V4‐V5 dataset, for (C) phyla/proteobacterial classes and (D) lineages/genera. Groups that were significantly higher in the V4 dataset are indicated by downward arrows while groups that were significantly higher in the V4‐V5 dataset are indicated by upward arrows. Significance was determined at p < 10^−6^ using a Wald test comparing log2 fold change.
**Fig. S2.** Depth profiles of temperature, dissolved oxygen, and fluorescence for stations sampled in each lake in 2012 (green) and 2013 (pink). Colour shades correspond to specific stations; dotted vs. solid lines correspond to spring and summer profiles, respectively.
**Fig. S3.** Depth profiles of NO_2_^−^ + NO_3_^−^, total dissolved phosphorus (TDP), and SiO_4_/SiO_3_ as Silica, collected in 2012 and 2013 by the US EPA during spring and summer surveys. Colours correspond to lake (or basin within Lake Erie); shape corresponds to year.
**Fig. S4.** Principal coordinate analysis showing community similarity among summer samples, based on pairwise Bray–Curtis similarity. Each panel shows the same points coloured by different factors. (A) Points denote lake (colour) and depth code (shape). Arrows illustrate correlations between community composition and environmental factors calculated by envfit with R^2^ values in parentheses. All factors shown were significantly correlated with variation in microbial communities at p < 0.001 except for total phosphorus (p = 0.002) and turbidity (p = 0.005). For additional plots, points are coloured by: (B) percent of surface light available, calculated based on Kd490, (C) temperature, (D) chlorophyll *a*, (E) total oxidized nitrogen.
**Fig. S5.** Depth, light, and temperature correlate with community similarity. Each panel shows the relationship between principal coordinate analysis axis 1 (from Fig. S4) and (A) depth, (B) percent of surface light available, calculated based on Kd490, (C) temperature.
**Fig. S6.** Principal coordinate analysis showing community similarity among summer surface samples, based on pairwise Bray–Curtis similarity. Arrows illustrate correlations between community composition and environmental factors calculated by envfit with R^2^ values in parentheses. All factors shown were significantly correlated with variation in microbial communities at p < 0.001 except for conductivity (p = 0.004) and dissolved oxygen (p = 0.005).
**Fig. S7.** Abundance vs. prevalence, shown by depth layer in summer. Each panel shows the maximum relative abundance (percent of sequences) of each oligotype as a function of the number of samples where that oligotype was detected, for (A) surface samples, (B) deep chlorophyll layer, and (C) deep bottom layer samples. Dotted lines indicate relative abundances of 1% and 10% and prevalence values of 10%, 80%, and 100% of samples. Oligotypes are colour‐coded by phylum/class and symbol sizes represent the median relative abundance for each oligotype (when detected). Oligotypes that achieved a high maximum relative abundance in multiple depth layers are numbered.
**Fig. S8.** Distribution of abundant oligotypes in surface and deep bottom samples during summer stratification. Frequency of abundance is reported as the number of samples where the relative abundance of each oligotype exceeds 1% of the total number of sequences (Bacteria + Archaea). Symbol colour corresponds to phylum; labels are included for high frequency oligotypes, identifying them to freshwater tribe where possible. Dotted grey lines show sample frequencies 10% and 80%.
**Fig. S9.** Relative abundance within acI‐B1 of a conspicuous minor oligotype as a function of temperature. This oligotype is coloured blue in Fig. 6. Sampling points are colour coded by lake; symbol shape denotes sampling depth; open vs. closed symbols signify samples collected in spring and summer, respectively.

**Table S1** Sample information, including station name and location, sample depth and depth code, and collection date.
**Table S2.** Phyla and proteobacterial classes that were significantly different when samples were characterized using the 16S rRNA V4 region vs. V4‐V5 region primers, based on a Wald test of log2 fold change.
**Table S3.** Oligotypes that were significantly different when samples were characterized using the 16S rRNA V4 region vs V4‐V5 region primers based on a Wald test of log2 fold change.
**Table S4.** Results of Permutational Multivariate Analysis of Variance comparing the effects of depth, lake, and year on microbial community composition characterized using primers targeting the 16S rRNA V4‐V5 region and the 16S rRNA V4 region.
**Table S5.** Phyla and proteobacterial classes that are significantly enriched in surface or deep‐water samples based on a Wald test of log2 fold change.
**Table S6.** Sequence distribution of manual oligotypes of four ubiquitous, abundant tribes and corresponding PERMANOVA results testing for effects of lake and depth.
