# Supplementary material for: Microbial communities of the Laurentian Great Lakes reflect connectivity and local biogeochemistry
Source: Environ Microbiol. 2019 Dec 2;22(1):433–46. doi: 10.1111/1462-2920.14862 (PMC6973239; doi:10.1111/1462-2920.14862)
Supplement: Supplementary file 1 — Figure S1 Comparing two primer sets for 16S rRNA gene amplicon sequencing. (A, B) Rank abundance curves for all Great Lakes samples, based on (A) V4 region primers or (B) V4‐V5 region primers. Points are coloured by phylum/proteobacterial class. (C, D) Relative abundance estimates in the V4 data set vs. the V4‐V5 dataset, for (C) phyla/proteobacterial classes and (D) lineages/genera. Groups that were significantly higher in the V4 dataset are indicated by downward arrows while groups that were significantly higher in the V4‐V5 dataset are indicated by upward arrows. Significance was determined at p < 10−6 using a Wald test comparing log2 fold change. Fig. S2. Depth profiles of temperature, dissolved oxygen, and fluorescence for stations sampled in each lake in 2012 (green) and 2013 (pink). Colour shades correspond to specific stations; dotted vs. solid lines correspond to spring and summer profiles, respectively. Fig. S3. Depth profiles of NO2 − + NO3 −, total dissolved phosphorus (TDP), and SiO4/SiO3 as Silica, collected in 2012 and 2013 by the US EPA during spring and summer surveys. Colours correspond to lake (or basin within Lake Erie); shape corresponds to year. Fig. S4. Principal coordinate analysis showing community similarity among summer samples, based on pairwise Bray–Curtis similarity. Each panel shows the same points coloured by different factors. (A) Points denote lake (colour) and depth code (shape). Arrows illustrate correlations between community composition and environmental factors calculated by envfit with R2 values in parentheses. All factors shown were significantly correlated with variation in microbial communities at p < 0.001 except for total phosphorus (p = 0.002) and turbidity (p = 0.005). For additional plots, points are coloured by: (B) percent of surface light available, calculated based on Kd490, (C) temperature, (D) chlorophyll a, (E) total oxidized nitrogen. Fig. S5. Depth, light, and temperature correlate with community similarity. E [file EMI-22-433-s001.docx]

**Supplemental Figures**


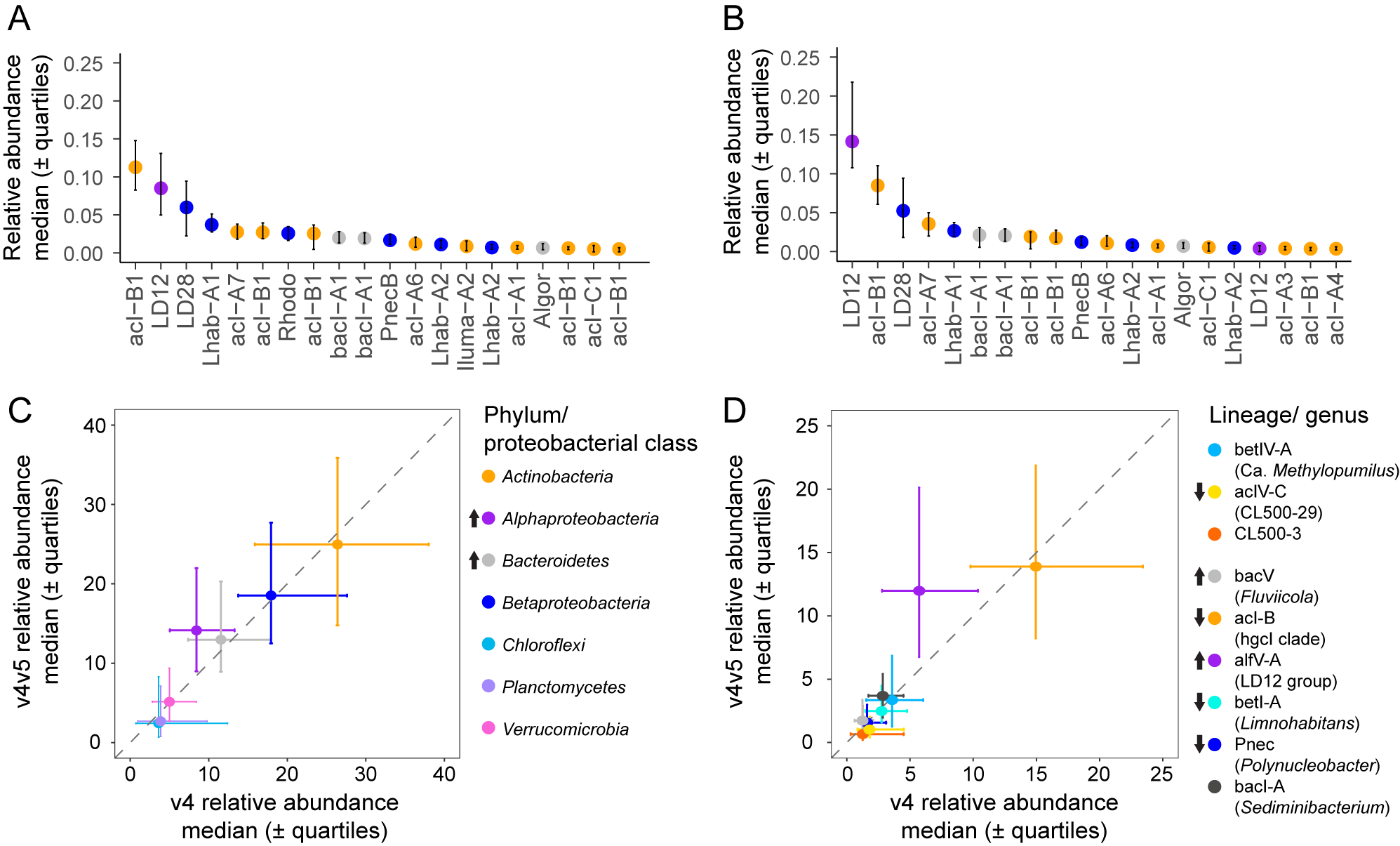


Figure S1. Comparing two primer sets for 16S rRNA gene amplicon sequencing. (A, B) Rank abundance curves for all Great Lakes samples, based on (A) V4 region primers or (B) V4-V5 region primers. Points are colored by phylum/proteobacterial class. (C, D) Relative abundance estimates in the V4 dataset *vs*. the V4-V5 dataset, for (C) phyla/proteobacterial classes and (D) lineages/genera. Groups that were significantly higher in the V4 dataset are indicated by downward arrows while groups that were significantly higher in the V4-V5 dataset are indicated by upward arrows. Significance was determined at p<10^-6^ using a Wald test comparing log2 fold change.

Figure S2. Depth profiles of temperature, dissolved oxygen, and chlorophyll *a* fluorescence for stations sampled in each lake in 2012 (green) and 2013 (pink). Color shades correspond to specific stations; dotted *vs.* solid lines correspond to spring and summer profiles, respectively.


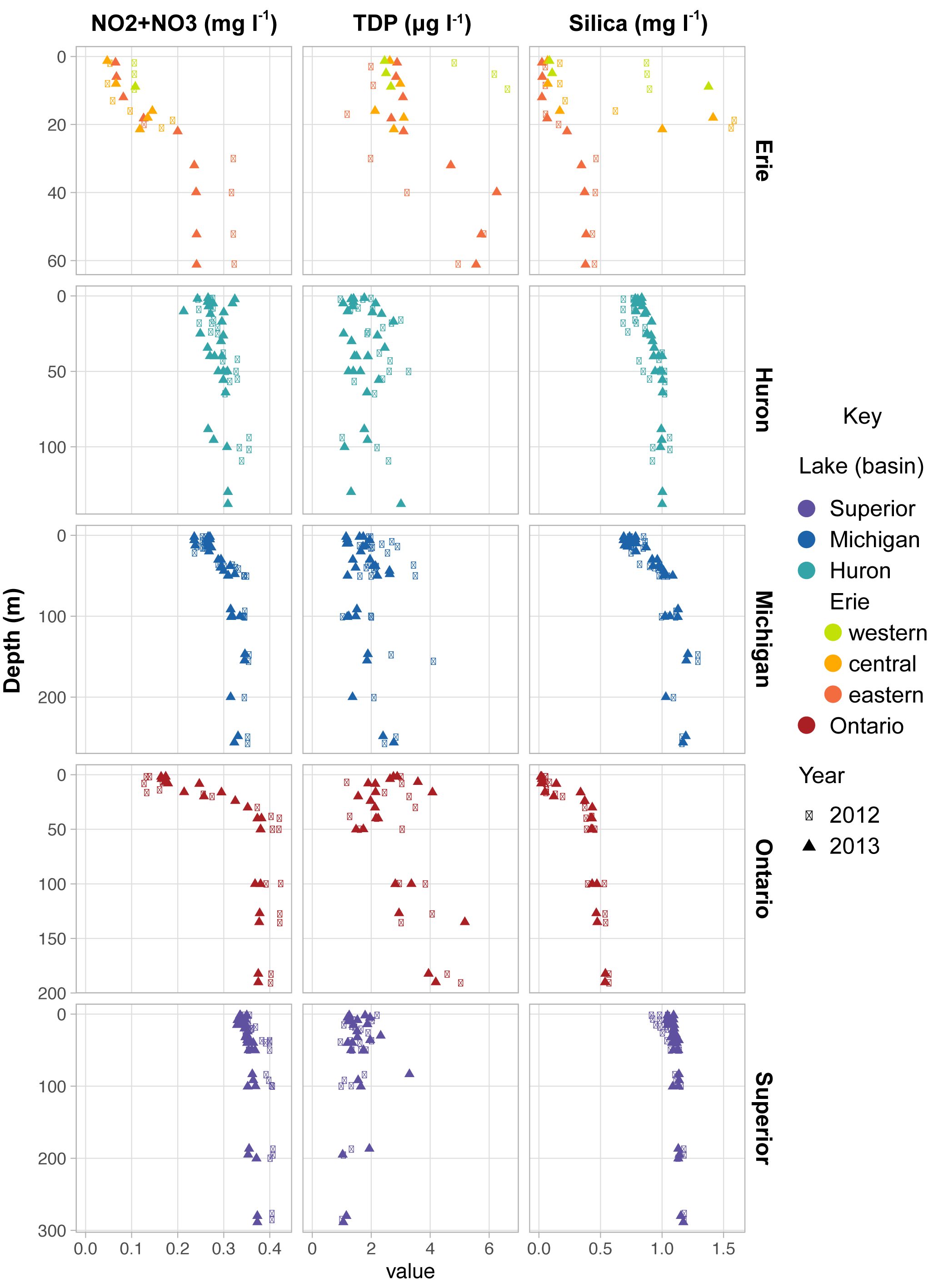


Figure S3. Depth profiles of NO_2_^-^+NO_3_^-^, total dissolved phosphorus (TDP), and SiO_4_/SiO_3_ as Silica, collected in 2012 and 2013 by the US EPA during spring and summer surveys. Colors correspond to lake (or basin within Lake Erie); shape corresponds to year.


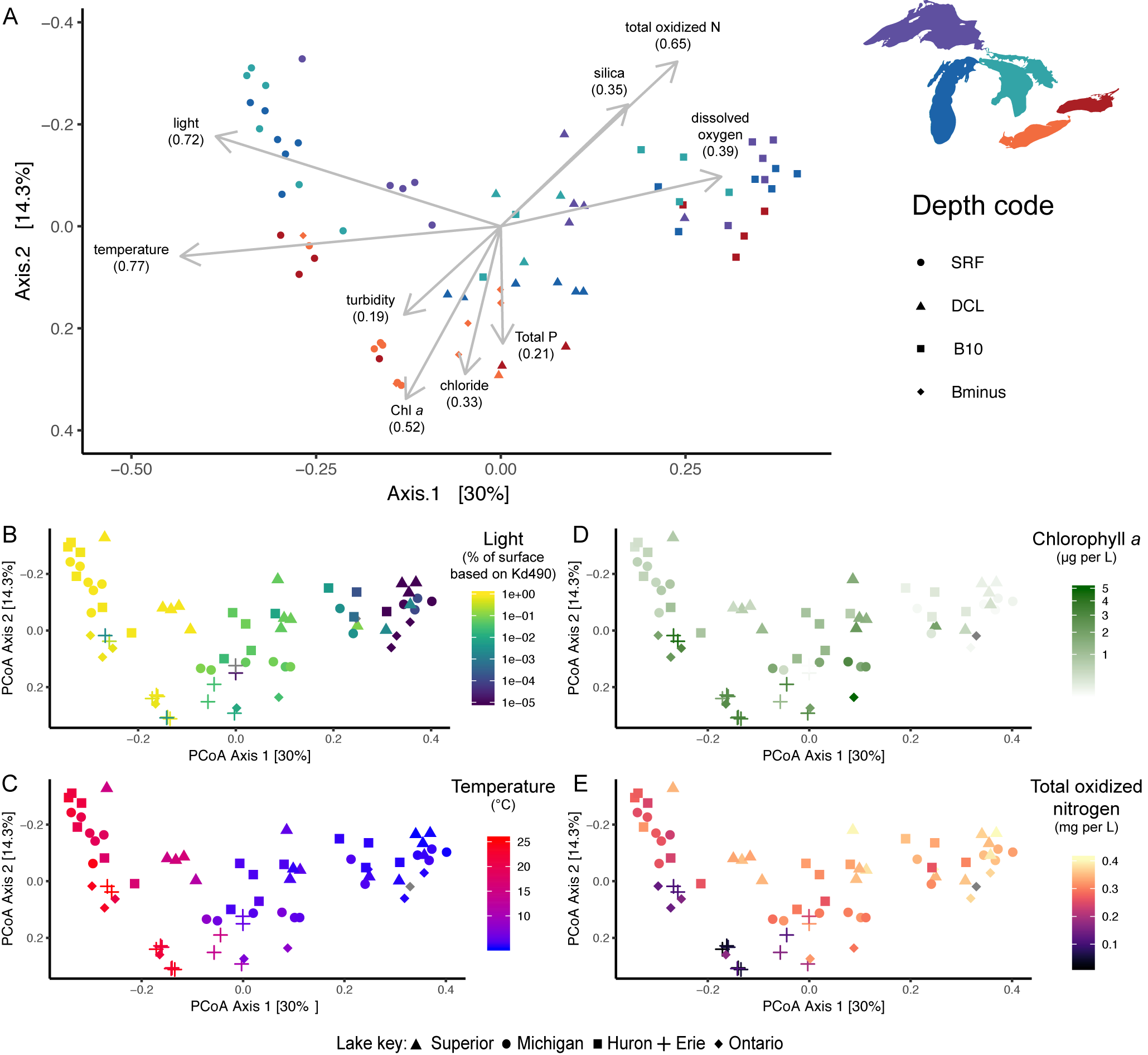


Figure S4. Principal coordinate analysis showing community similarity among summer samples, based on pairwise Bray-Curtis similarity. Each panel shows the same points colored by different factors. (A) Points denote lake (color) and depth code (shape). Arrows illustrate correlations between community composition and environmental factors calculated by envfit with R^2^ values in parentheses. All factors shown were significantly correlated with variation in microbial communities at p<0.001 except for total phosphorus (p=0.002) and turbidity (p=0.005). For additional plots, points are colored by: (B) percent of surface light available, calculated based on Kd490, (C) temperature, (D) chlorophyll *a*, (E) total oxidized nitrogen.


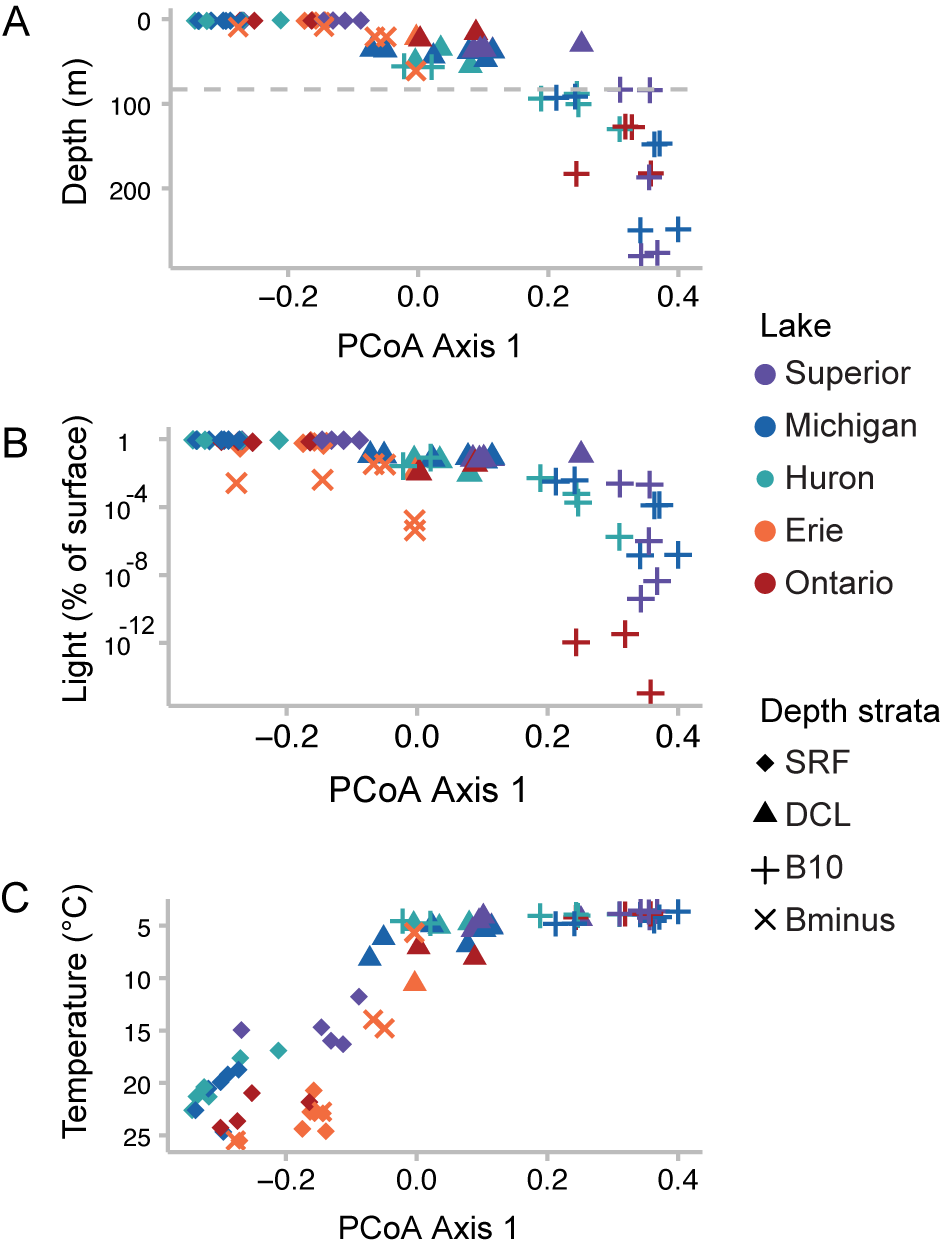


Figure S5. Depth, light, and temperature correlate with community similarity. Each panel shows the relationship between principal coordinate analysis axis 1 (from Fig. S4) and (A) depth, (B) percent of surface light available, calculated based on Kd490, (C) temperature.


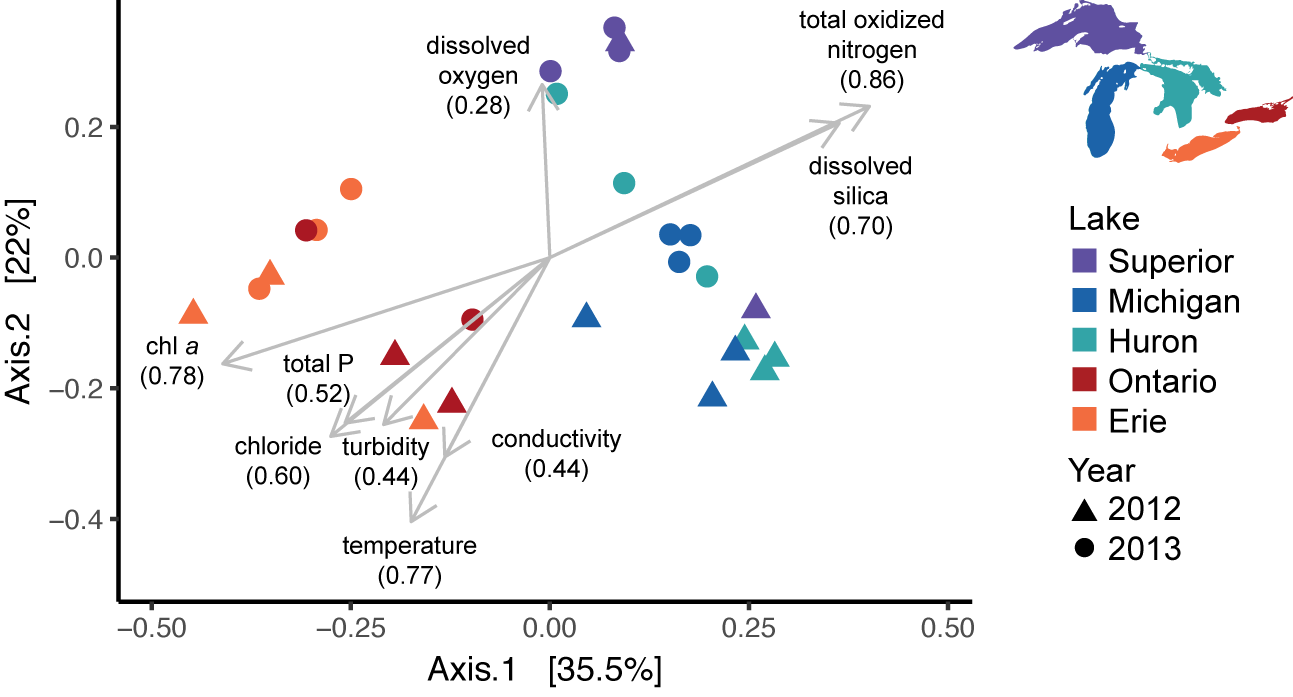


Figure S6. Principal coordinate analysis showing community similarity among summer surface samples, based on pairwise Bray-Curtis similarity. Arrows illustrate correlations between community composition and environmental factors calculated by envfit with R^2^ values in parentheses. All factors shown were significantly correlated with variation in microbial communities at p<0.001 except for conductivity (p=0.004) and dissolved oxygen (p=0.005).


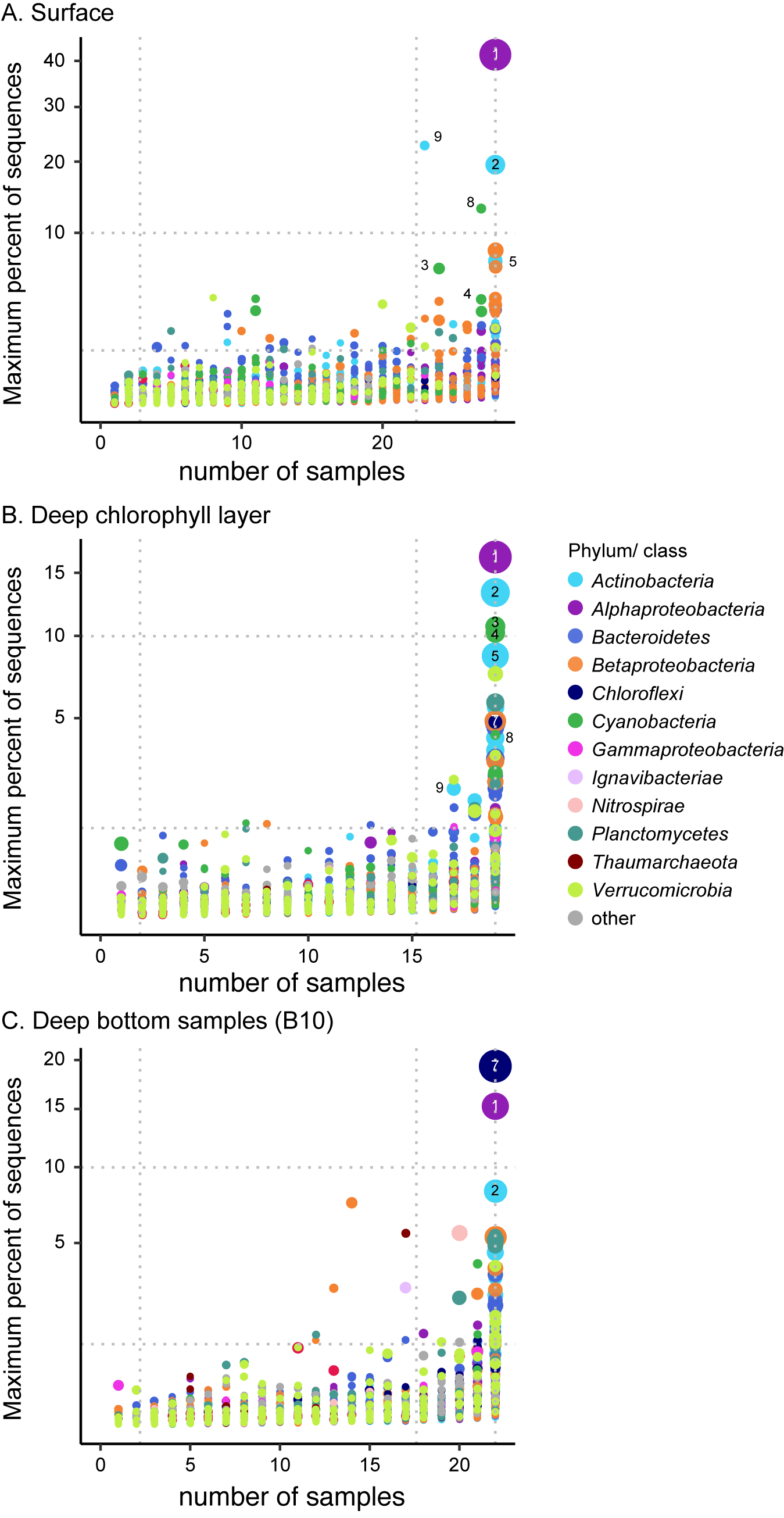


Figure S7. Abundance vs. prevalence, shown by depth layer in summer. Each panel shows the maximum relative abundance (percent of sequences) of each oligotype as a function of the number of samples where that oligotype was detected, for (A) surface samples, (B) deep chlorophyll layer, and (C) deep bottom layer samples. Dotted lines indicate relative abundances of 1% and 10% and prevalence values of 10%, 80%, and 100% of samples. Oligotypes are color-coded by phylum/class and symbol sizes represent the median relative abundance for each oligotype (when detected). Oligotypes that achieved a high maximum relative abundance in multiple depth layers are numbered.


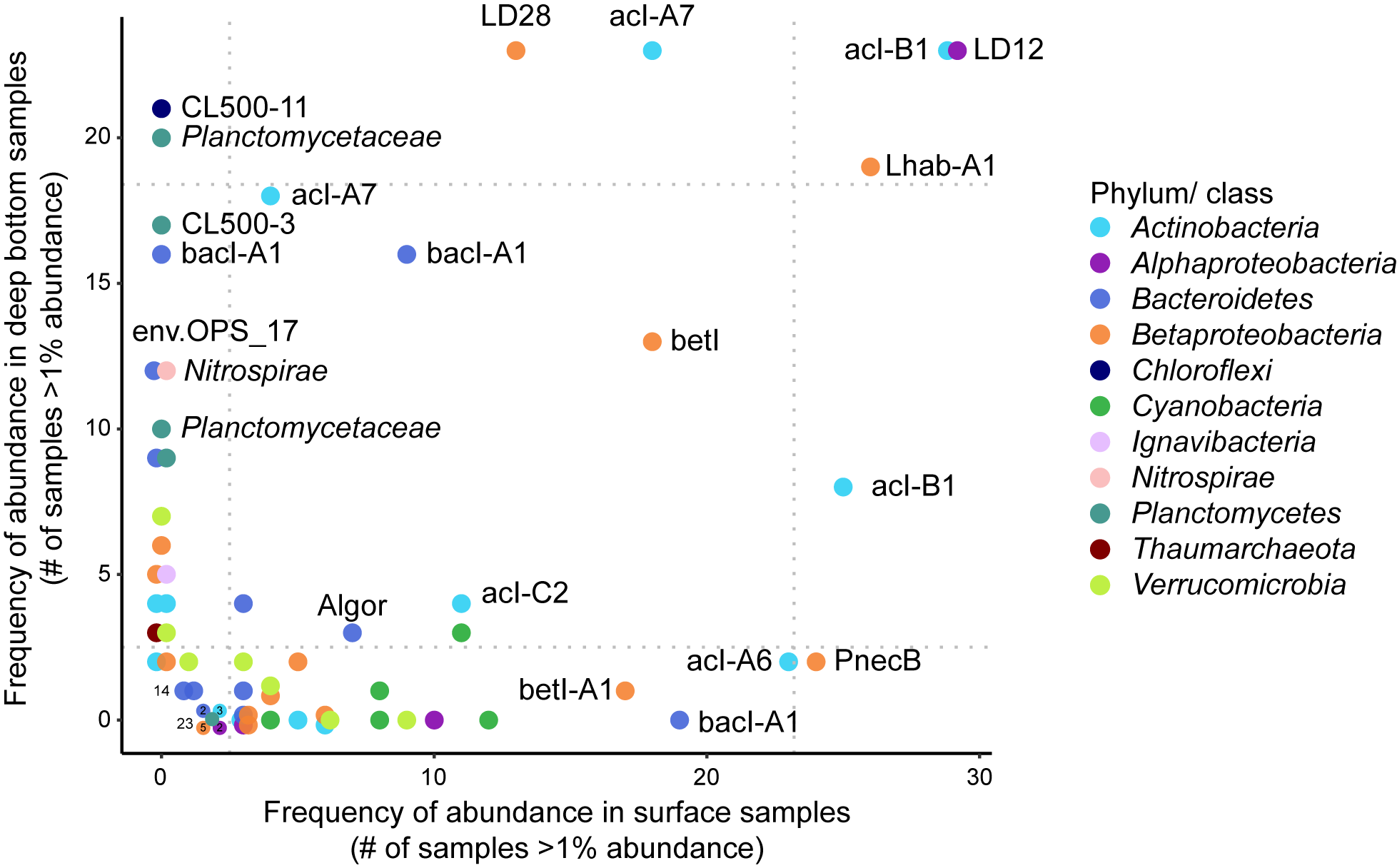


Figure S8. Distribution of abundant oligotypes in surface and deep bottom samples during summer stratification. Frequency of abundance is reported as the number of samples where the relative abundance of each oligotype exceeds 1% of the total number of sequences (Bacteria + Archaea). Symbol color corresponds to phylum; labels are included for high frequency oligotypes, identifying them to freshwater tribe where possible. Dotted grey lines show sample frequencies 10% and 80%.


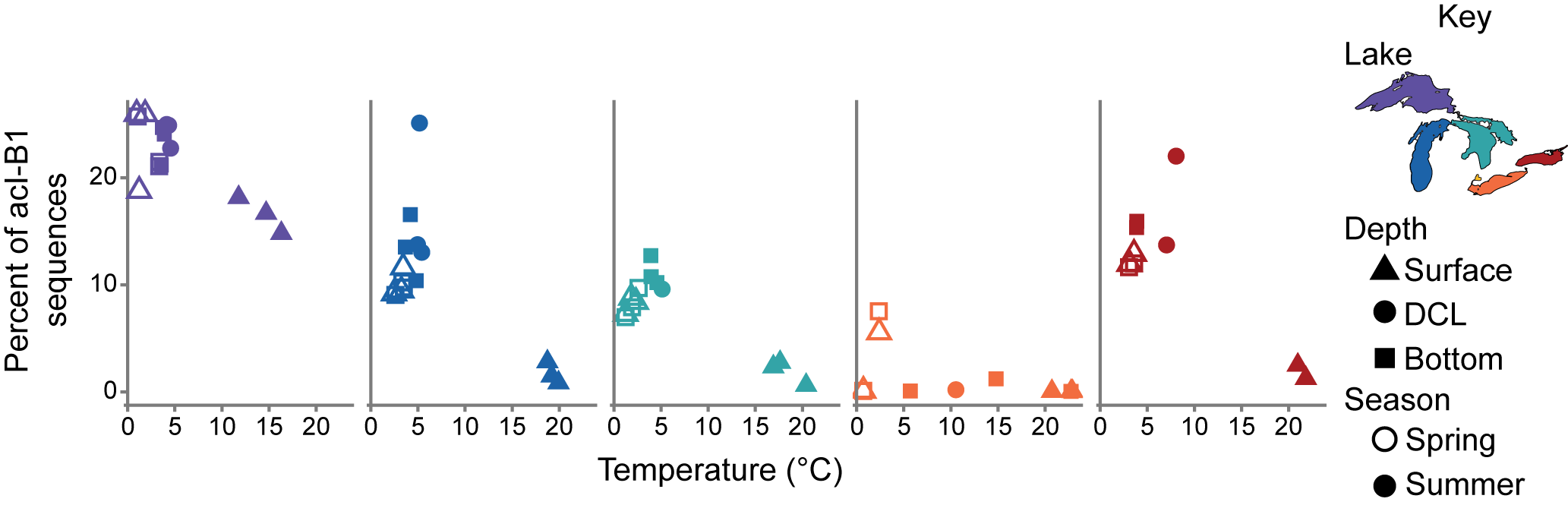


Figure S9. Relative abundance within acI-B1 of a conspicuous minor oligotype as a function of temperature. This oligotype is colored blue in Fig. 6. Sampling points are color coded by lake; symbol shape denotes sampling depth; open vs closed symbols signify samples collected in spring and summer, respectively.
